# Circulating and extracellular vesicle-derived microRNAs as biomarkers in bone-related diseases

**DOI:** 10.3389/fendo.2023.1168898

**Published:** 2023-05-24

**Authors:** Julika Huber, Michael T. Longaker, Natalina Quarto

**Affiliations:** ^1^ Hagey Laboratory for Pediatric Regenerative Medicine, Stanford University School of Medicine, Stanford, CA, United States; ^2^ Division of Plastic and Reconstructive Surgery, Stanford University School of Medicine, Stanford, CA, United States; ^3^ Department of Surgery, Stanford University School of Medicine, Stanford, CA, United States; ^4^ Department of Plastic Surgery, University Hospital Bergmannsheil Bochum, Bochum, Germany; ^5^ Institute for Stem Cell Biology and Regenerative Medicine, Stanford University School of Medicine, Stanford, CA, United States

**Keywords:** microRNA, biomarker, bone, osteosarcoma, osteoporosis, extracellular vesicles, exosomes

## Abstract

MicroRNAs (miRNA) are small non-coding RNA molecules that regulate posttranscriptional gene expression by repressing messengerRNA-targets. MiRNAs are abundant in many cell types and are secreted into extracellular fluids, protected from degradation by packaging in extracellular vesicles. These circulating miRNAs are easily accessible, disease-specific and sensitive to small changes, which makes them ideal biomarkers for diagnostic, prognostic, predictive or monitoring purposes. Specific miRNA signatures can be reflective of disease status and development or indicators of poor treatment response. This is especially important in malignant diseases, as the ease of accessibility of circulating miRNAs circumvents the need for invasive tissue biopsy. In osteogenesis, miRNAs can act either osteo-enhancing or osteo-repressing by targeting key transcription factors and signaling pathways. This review highlights the role of circulating and extracellular vesicle-derived miRNAs as biomarkers in bone-related diseases, with a specific focus on osteoporosis and osteosarcoma. To this end, a comprehensive literature search has been performed. The first part of the review discusses the history and biology of miRNAs, followed by a description of different types of biomarkers and an update of the current knowledge of miRNAs as biomarkers in bone related diseases. Finally, limitations of miRNAs biomarker research and future perspectives will be presented.

## 1 Introduction

MicroRNAs (miRNAs) are small non-coding RNA molecules, about 19-24 nucleotides in length, that regulate posttranscriptional gene expression by targeting messengerRNAs (mRNAs) and thus repress or alter the translational process (1, 2). Each miRNA targets more than 100 genes and plays a role in multiple signaling pathways and biological processes (2). They can be secreted into extracellular fluids and act as messengers to mediate cell-cell communication (2).

The first miRNA-mRNA interaction has been described by Victor Ambros and Gary Ruvkun in 1993 (3, 4). They discovered *lin-4* binding to the 3’ untranslated region of *lin-14* mRNA and thereby regulating the level of LIN-14 protein, necessary for temporal development in *Caenorhabditis elegans* (3, 4). Since then, miRNAs have also been detected in flies, fish and mammals and are generally conserved amongst species (2, 5–10). With the discovery of miRNAs and target prediction facilitated by whole genome sequencing and bioinformatic approaches, researchers shifted their research interests towards functional characterizations of miRNA-mRNA interactions (11).

Given that miRNAs are tissue-specific and their expression patterns reflect cellular physiological processes, their importance in the context of human diseases became more and more apparent (1, 11). For instance, miRNAs can act as tumor suppressors or oncogenes, regulate cellular proliferation and apoptosis, modulate intracellular signaling and immune response (1, 11). Specifically, in oncologic diseases, miRNAs are involved in cell apoptosis, invasion and metastasis and disease progression and have diagnostic, prognostic and therapeutic use (11–20). Moreover, miRNAs have also been implied in viral disease (11, 21, 22), neurodegenerative disease (11, 23, 24) and immune disease (25, 26).

MiRNAs are also known to play an important role in epigenetic regulation of osteogenesis (27). By targeting key osteogenic regulators, *e.g.* runt-related transcription factor 2 (*RUNX2)*, they can enhance or repress osteogenesis (27–37). Other miRNAs act by targeting genes and receptors of osteogenic signaling pathways, such as the Wnt- or Bone morphogenetic protein (BMP)-signaling pathway (38–48). Additionally, several miRNAs also regulate osteoclast differentiation and osteoblast-osteoclast communication (49, 50).

**Figure 1:**
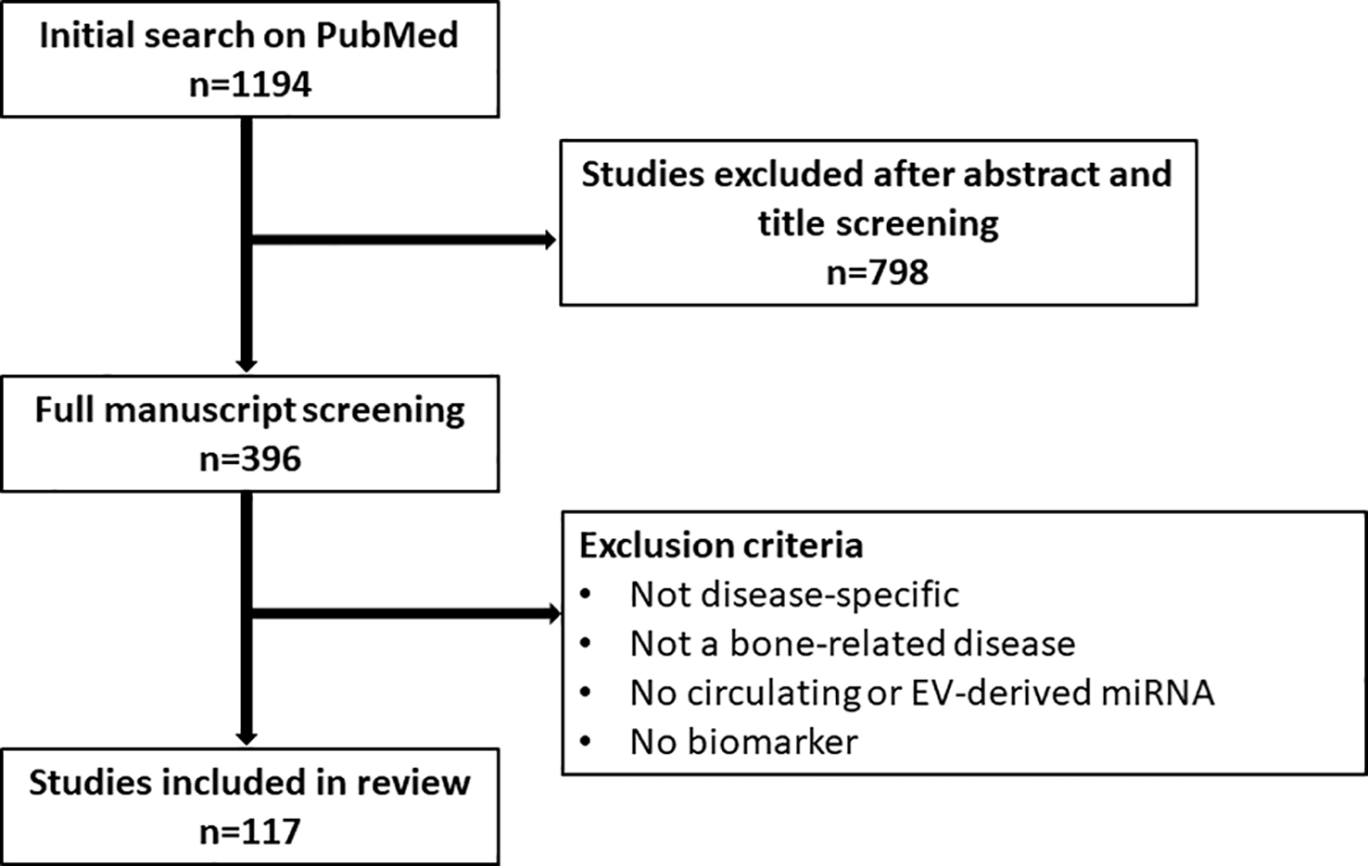
Flow chart depicting the screening process for studies included in the review.

Considering that miRNAs are reflective of certain cellular and molecular processes, changes in their expression level are a useful indicator of disease, thus making them ideal biomarkers for diagnostic, predictive and prognostic purposes (1, 27, 51, 52). Additionally, miRNAs are packaged into extracellular vesicles (EVs) and released into the bloodstream, protected from degradation (27, 51). These circulating miRNAs and EV-miRNAs are easily harvested by liquid biopsy and thus represent an accessible tool to study miRNA signatures as biomarkers, circumventing the need for invasive tissue biopsy specifically in bone-related diseases (27, 51). Moreover, EV-miRNAs can also be harvested locally before being released into the bloodstream, *e.g.* in bile or cerebrospinal fluid, or can be linked to their tissue of origin based on detection of surface marker proteins, thus allowing for better diagnostic specificity and sensitivity (53). In this review, we highlight the role of circulating miRNAs as biomarkers in bone-related diseases, focusing on osteoporosis and osteosarcoma. To this end, a comprehensive literature search on PubMed has been performed. We applied the following search query and a filter for English language: (((((((((exosomes) OR (microvesicles)) OR (extracellular vesicles)) OR (circulating)) AND (miRNA)) OR (microRNA)) OR (miR)) AND (bone)) AND (biomarker)) NOT (Review). After an initial screening of titles and abstracts (n=1194), a more in-depth screening of the manuscript text was performed among the included 396 papers. Studies only discussing miRNA in a general osteogenesis context, but not disease-specific or studies not investigating a bone-related disease were excluded. Additionally, studies that did not discuss circulating or EV-derived miRNA or did not discuss their biomarker potential were excluded. The following review encompasses the remaining 117 studies (**Figure 1**).

## 2 Disease biomarkers

According to the Biomarkers, EndpointS, and other Tools (BEST)- glossary by the FDA-NIH Joint Leadership Council biomarkers are “a defined characteristic that is measured as an indicator of normal biological processes, pathogenic processes, or biological responses to an exposure or intervention, including therapeutic interventions”. They can be categorized into 7 different types, including susceptibility/risk, diagnostic, monitoring, prognostic, predictive, response and safety biomarker.

The susceptibility biomarker is a risk indicator for developing a disease before the onset of disease. In clinical practice, it is helpful to inform preventative care, such as making lifestyle or dietary changes or increasing the frequency of screening exams. Well known examples of susceptibility biomarkers are the Breast Cancer Gene 1/2 (*BRCA1/2)* mutations (54, 55), that indicate the likelihood of developing breast and ovarian cancer, and low-density lipoprotein cholesterol levels (56), that are accompanied by an increased risk for development of cardiovascular disease.

A diagnostic biomarker confirms a disease or a specific subtype of a disease, which may guide treatment decisions or potential enrollment in clinical trials. Common examples include the Glomerular filtration rate (GFR) to identify patients with chronic kidney disease (57), sweat chloride to identify individuals with cystic fibrosis (58) or HbA1c to diagnose diabetes mellitus (59).

A monitoring biomarker assesses the status of a disease or treatment response and is measured repeatedly. For instance, they are useful for assessment of disease progression, disease recurrence, changes in severity status of disease. Measurements are usually taken during defined periods of the disease, such as from diagnosis to treatment, *e.g.* to evaluate progression rate, during intervention, to assess treatment response or after treatment, to monitor recurrence. Common examples include Prostate-specific antigen (PSA), which is used to monitor disease status in patients with prostate cancer (60, 61) and International normalized ration (INR) or prothrombin time (PT) to evaluate anticoagulative treatment response (62).

A prognostic biomarker indicates the likelihood of a clinical event, disease recurrence or progression of disease, such as metastasis or overall survival, after onset of disease. They are measured at a defined baseline and are dependent on clinical setting, *e.g.* ongoing background treatment or stage of disease, and the endpoint of interest. They can inform decisions about treatment plans, for instance, plasma fibrinogen is used to identify patients with high risk for exacerbation of chronic obstructive pulmonary disease (63).

A predictive biomarker identifies patients who are more likely to have a defined outcome from a treatment and are useful for treatment selection. For instance, they can be characteristic to the patient’s constitution, *e.g.* a genetic marker, expression level of a specific protein in tissues or serum or mutations in tumor. On the other hand, predictive biomarkers can be dependent on the drug, which is often defined based on empiric evidence and pathophysiology of the drug. For example, in non-small cell lung cancer, squamous differentiation is predictive of a negative response to pemetrexed treatment (64). It is often difficult to distinguish between a prognostic and a predictive biomarker. In certain cases, a biomarker can also simultaneously be both, prognostic and predictive.

A response biomarker, *e.g.* a pharmacodynamic biomarker, indicates that a biological response has occurred after exposure to a treatment. A common example is circulating B lymphocytes to assess response to a B-lymphocyte stimulator in patients with systemic lupus erythematosus (65). In a clinical context, response biomarkers help guide decisions regarding dosing or continuation of a treatment, *e.g.* HbA1c to evaluate the response to antihyperglycemic agent in diabetes mellitus patients. Treatment plans can be modified according to the level of response.

Last, a safety biomarker is an indicator for toxicity of a treatment, which can be measured before or after treatment start and can predict, confirm or evaluate the extent of the adverse effect. Measurements are often taken repeatedly, to ensure adequate management of toxicity and adjustment of treatment plans, *e.g.* serum creatinine to monitor nephrotoxic effects of certain drugs (66). Additionally, if taken before start of the treatment, safety biomarkers can identify patients, who should not be given a specific treatment due to safety risk, such as deficiencies of metabolizing enzymes. An example includes the HLA-B* 1502 genotype to identify patients with an increased risk of developing Steven-Johnson syndrome upon treatment with carbamazepine (67).

**Figure 2:**
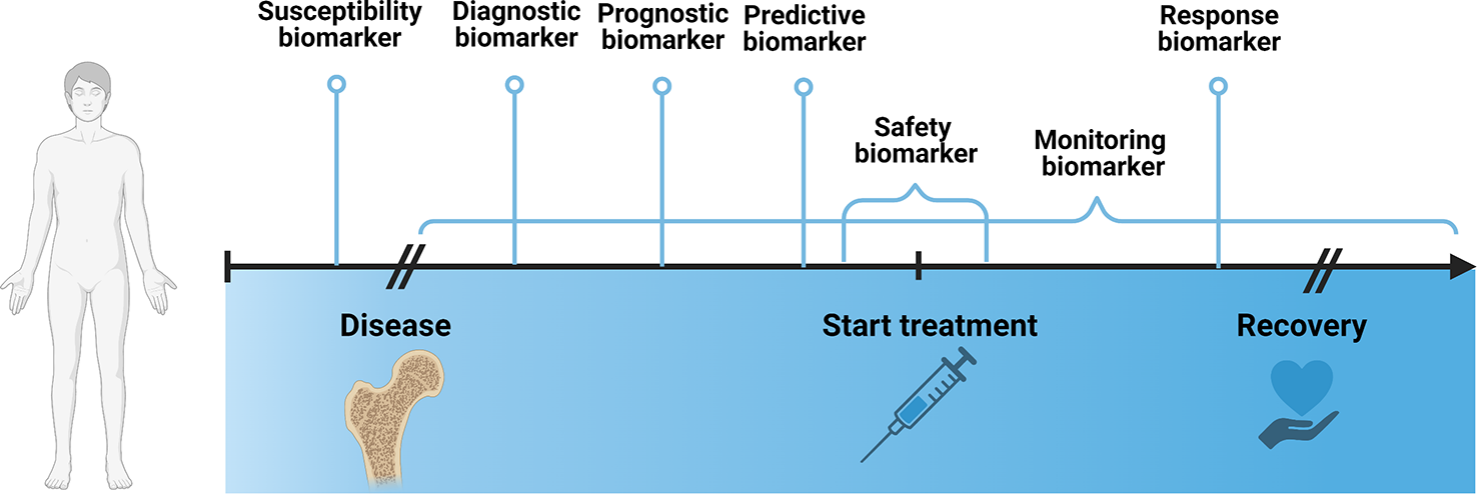
Schematic depicting different biomarker types in the context of disease development over time. Created with BioRender.com.

**Figure 2** establishes a timeline from before the onset of disease until after discovery and marks the timepoints at which a specific biomarker is measured.

In the following, we aim to review current knowledge about miRNAs as biomarkers for bone-related diseases, specifically in osteoporosis and osteosarcoma.

## 3 MiRNA biomarkers in osteoporosis

Osteoporosis is the most common bone disease and affects over 10 million people in the United States and over 200 million women worldwide (68). An increase in osteoclast activity and a decrease in osteoblast activity leads to an imbalance in bone homeostasis and causes bone loss. The resulting decrease in bone density and bone mass results in bone fragility and increased risk of fracture (52, 68–70). In 2006, an estimated 8.9 million of osteoporotic fractures were recorded worldwide and 1 in 3 women and 1 in 5 men will experience a fracture after the age of 50 years (71, 72). Risk factors include postmenopausal estrogen deficiency, low physical activity, smoking, hormonal factors, genetic factors, nutrition and the use of specific drugs, *e.g.* glucocorticoids. Given our aging population and changing lifestyle habits, osteoporosis-related morbidity is expected to increase, which represents a major burden for the global health system (68, 72, 73). Thus, early identification of individuals at high risk for osteoporosis is important to allow for preventative care in the form of lifestyle interventions or pharmacologic treatments (68, 74). Current diagnostic methods include bone mineral density (BMD) measurements by dual-energy X-ray absorptiometry (DXA), which is recommended for all women 65 years and older (68). Bone turnover markers, such as alkaline phosphatase (ALP), osteocalcin (OCN) or parathyroid hormone (PTH), are valuable in risk assessment in combination with BMD measurements (68, 74). The US Preventive Services Task force (USPSTF) suggests questionnaire-based screening tools, *e.g.* Fracture Risk Assessment (FRAX), Osteoporosis Risk Assessment Instrument (ORAI) or Osteoporosis Index of Risk (OSIRIS), to assess increased osteoporotic risk in postmenopausal women younger than 65 years old.

However, DXA is expensive and comes with radiation exposure and bone turnover markers are unreliable, thus better screening methods are highly sought after. In the past decade, miRNA signatures have emerged as potential biomarkers for the early diagnosis of osteoporosis and risk prediction for fragility fractures.

Multiple authors have explored the potential of individual or a panel of miRNA as diagnostic biomarkers in postmenopausal osteoporosis (PMO) (52, 69, 75–84), in general patient cohorts including PMO and idiopathic osteoporosis (85–93) and in specific secondary osteoporosis cases, *e.g.* severe childhood-onset osteoporosis in patients with mutations in the Plastin 3 (*PLS3*) gene (94). Among the most investigated miRNAs are members of the miR-21-, miR-23-, miR-320-, miR-203- and miR-19-families. miR-203 is a known suppressor of bone formation by negatively regulating *BMP*-2 and distal-less homeobox 5 (*DLX5*) which are activators of key transcription factors for osteoblast differentiation like runt *RUNX2* and Osterix (*SP7*) (79, 80, 94). miR-23 is also known to negatively regulate *RUNX2* (92). miR-21 is one of the most studied microRNAs. Among its direct targets are members of the Transforming Growth Factor ß (TGFß)- and BMP-signaling pathways, *RUNX2*, osteocalcin, osteopontin, phosphatase and tensin homolog (*PTEN)* and *SMAD7* (95). miR-21 is also involved in osteoclastogenesis by targeting osteoclast suppressor programmed cell death protein 4 (*PDCD4*) and osteoclast marker cathepsin K (*CTSK*) and receptor activator of nuclear factor ĸB ligand (*RANKL*) (92, 95), promoting osteoclast differentiation. miR-19b directly targets *PTEN* resulting in an increase of *RUNX2* and phosphorylation of AKT (protein kinase B) and thus enhancing osteoblastogenesis (96, 97). Targets of miR-320 include *BMP2*, *CTNNB1* (ß-catenin) and *RUNX2*, promoting osteoblast differentiation (98) (**Table 1**).

**Table 1:** MiRNA biomarkers in osteoporosis.

Bone-related disease	Author	MiRNA	Target genes of interest	Type of biomarker	Species	MiRNA source
**Osteoporosis**	Al-Rawaf et al. (69)	miR-148a miR-122-5p		Diagnostic biomarker for PMO vs controls	Human	Serum
	Baloun et al. (52)	miR-1278 miR-24-1-5p miR-422a		Diagnostic biomarker for PMO vs controls	Human	Serum
	Bedene et al. (75)	miR-148a-3p	*MAFB, PPAR, WNT1*	Diagnostic biomarker for PMO vs controls	Human	Plasma
	Chen et al. (76)	miR-21-5p miR-23a-3p miR-125-5p	*PDCD4, ASL, EIF4E3* (miR-21) *RUNX2* (miR-23) *PDGF* (miR-125b)	Diagnostic biomarker for PMO vs controls	Human	Serum
	Ciuffi et al. (85)	miR-23a-3p miR-21-5p miR-320a-3p		Diagnostic biomarker for osteoporosis vs osteopenia vs healthy controls (miR-23a-3p, miR-21a-5p) Diagnostic biomarker for osteoporosis with fracture vs osteoporosis without fracture (miR-320a-3p)	Human	Serum
	Ding et al. (77)	Panel: miR-194-5p and five medical items (weight, age, left ventricular end systolic diameter, alanine aminotransferase, urine epithelial cell count)		Diagnostic biomarker for PMO vs osteopenia vs healthy controls	Human	Plasma
	Ding et al. (86)	miR-100		Diagnostic biomarker for osteoporosis vs healthy control	Human	Plasma
	Garg et al. (99)	miR-23b-3p miR-140-3p miR-21-5p miR-122-5p miR-125-5p	*SMAD7, FGF18, SKP2, SPRY1/2, PDCD4, PTEN, RECK, GDF-5, SOX2, PLAP-1, ACVR2B* (miR-21-5p) *RUNX2, MRC2, CCND1, PTEN* (miR-23b-3p) *BMP2, IGF1R, RUNX2,SPARC, TSC22D3, VDR, PCP4* (miR-122-5p) *BMPR1B, TRAF6* (miR-125b-5p) *MCF2L, PTEN, BMP2* (miR-140-3p)	Diagnostic biomarker for osteoporotic hip fractures vs non-osteoporotic hip fractures	Human	Plasma
	Heilmeier et al. (97)	Panel: miR-203a, miR-31-5p, miR-19b-1-5p, BMD measurements	*FOS, RUNX2, SMAD1* (miR-203a) *PTEN, RUNX2* (miR-19b-1-5p) *FZD3, RUNX2, SP7, SATB2* (miR-31-5p)	Predictive biomarker for fracture risk in diabetic osteoporosis	Human	Serum
	Heilmeier et al. (100)	miR-382-3p, miR-188-3p, miR-942		Diagnostic biomarker for PM osteoporotic fragility fracture vs postmenopausal patients without fractures	Human	Serum
	Ismail et al. (78)	miR-208a-3p miR-155-5p miR-637	*ETS1, ACVR1* (miR-208a-3p) *SOCS1* (miR-155-5p) *SP7* (miR-637)	Diagnostic biomarker for PMO vs premenopausal osteoporosis vs healthy controls (miR-208a-3p) and for PMO vs healthy controls (miR-155-5p, miR-637)	Human	Serum
	Kerschan-Schindl et al. (79)	OsteomiR™ panel	*RUNX2, LRP5*, ß-catenin (miR-375) Osteocalcin, *CTX* (miR-550a-3p) *WNT10B* (miR-152-3p) BMP2, DLX5, Osteocalcin (miR-203a)	Diagnostic biomarker for PMO vs controls (miR-375) Diagnostic biomarker for postmenopausal osteoporotic fragility fractures vs osteoporosis without fracture (miR-203a)	Human	Serum
	Kocijan et al. (101)	Panel: miR-152-3p, miR-30e-5p, miR-324-3p, miR-19b-3p, miR-335-5p, miR-19a-3p, miR-550a-3p, miR-186-5p, miR-532-5p, miR-93-5p, miR-378-5p, miR-320a, miR-16-5p, miR-215-5p, let-7b-5p, miR-29b-3p, miR-7-5p, miR-365a-3p	*DKK1* (miR-152-3p, miR-335-3p) *LRP6* (miR-30e-5p) *BMP2* (miR-140-5p) *HDAC4, TGFß3, ACVR2A, CTNNBIP1, DUSP2* (miR-29b-2p)	Diagnostic biomarker for idiopathic and postmenopausal osteoporotic fractures vs non-osteoporotic fracture controls	Human	Serum
	Kocijan et al. (80)	miR-203a	*DLX5, RUNX2*	Diagnostic biomarker for PMO vs controls Monitoring biomarker for treatment response for zoledronate and teriparatide in PMO	Human	Serum
	Ladang et al. (102)	OsteomiR™ panel		Predictive biomarker for fragility fracture risk	Human	Serum
	Li et al. (81)	miR-21 miR-133a	*SPRY1* (miR-21)	Diagnostic biomarker for PM-osteopenia/osteoporosis vs controls	Human	Plasma
	Lincoln et al. (103)	miR-148a-3p	*WNT1, WNT10B, KDM6B, DNMT1, IGF1, MAFB*	Predictive biomarker for development of osteoporosis in patients with acute spinal cord injury	Human	Plasma
	Lu et al. (87)	miR-206	*HDAC4*	Diagnostic biomarker for osteoporosis vs control	Human	Serum
	Ma et al. (82)	miR-181c-5p miR-497-5p		Diagnostic biomarker for PMO vs controls Monitoring biomarker for bisphosphonate and calcitriol treatment response in PMO	Human	Serum
	Mäkitie et al. (94)	Panel: miR-93-3p, miR-532-3p, miR-133a-3p, miR-301b-3p, miR-181c-5p, miR-203a-3p, miR-590-3p	*WNT1, LRP6, PTEN* (miR-301b) *DLX5, RUNX2* (miR-203a-3p) *RANKL, MMP9, NF-ĸB, DKK1* (miR-218-5p) *DKK1* (miR-203)	Diagnostic biomarker for X-linked primary osteoporosis	Human	Serum
	Mandourah et al. (88)	miR-122-5p and/or miR-4516	*BMP2K, FSHB, IGF1R, RUNX2, SPARC, TSC22D3, TSC22D3, VDR* (both) *CNR2, ALPL, ANKH, ESR1, LRP6* (miR-122-5p) *CNR1, AR* (miR-4516)	Diagnostic biomarker for osteoporosis vs osteopenia vs control	Human	Serum and Plasma
	Meng et al. (83)	miR-194-5p	*COUP-TFII* TGFß-signaling pathway Wnt-signaling pathway	Diagnostic biomarker for PM- osteopenia/osteoporosis vs controls	Human	Whole blood
	Messner et al. (104)	miR-454-3p, miR-584-5p, miR-101-3p, miR-191-5p, miR-26-5p, miR-32-5p, miR-4508	Wnt- signaling pathway TGFß- signaling pathway	Monitoring biomarker for denosumab treatment response in PMO	Human	Serum
	Nakashima et al. (89)	miR-195 miR-150	*GIT1, BMP* (miR-195) *MMP14* (miR-150)	Diagnostic biomarker for osteoporosis vs control	Human	Serum
	Panach et al. (105)	miR-122-5p miR-125b-5p miR-21-5p		Diagnostic biomarker for osteoporotic bone fracture vs osteoarthritic control	Human	Serum
	Pepe et al. (90)	miR-1246 miR-1224-5p	Tetraspanin 5 (miR-1224-5p)	Diagnostic biomarker for osteoporosis vs osteopenia and control	Human	Plasma-exosomes
	Quillen et al. (98)	miR-197-3p miR-320a miR-320b miR-331-5p miR-423-5p	*FGB, IGFBP5, SOD1, SOD2* (miR-197-3p) *SOD2* (miR-331-5p) *GAPDH, MIF, MMP9, CTNNB1, RUNX2* (miR-320a) *GAPDH* (miR-423-5p) *BMP2* (miR-320b)	Susceptibility biomarker for bone loss	Baboon	Plasma
	Ramírez-Salazar et al. (91)	miR-140-3p miR-23b-3p	*AKT1, AKT2, AKT3, BMP2 FOXO3, GSK3B, IL6R, PRKACB, RUNX2, WNT5*	Diagnostic biomarker for osteopenia/osteoporosis vs control	Human	Serum
	Seeliger et al. (92)	Panel: miR-21, miR-23a, miR-24, miR-100, miR-125b	*PDCD4* (miR-21) *RUNX2* (miR-23a, miR-24) *BMPR2* (miR-100)	Diagnostic biomarker for osteoporosis vs control	Human	Serum
	Shi et al. (106)	miR-324-3p miR-766-3p miR-1247-5p miR-330-5p miR-3124-5p	*WNT8B, FZD2, CSNK1E, DVL1, RAC3* (miR-324-3p) *WNT1-A, FZD10, SENP2, VANGL1, WNT5B, PRKCA, NFATC2* (miR-776-3p) *WNT9B, DVL3, CSNK2A2, APC2, MYCBP2, RNC1* (miR-1247-5p) *WNT2B, FZD4, DVL3, PRKACA, APC2, BTRC, DVL1, PPP3CB* (miR-330-5p) *WNT10B, LRP6, CXXC4, CSNK2A1, GSK3B, CSN1A1L, FZD1, WNT5a, PLCB1, PRKCA* (miR-3124-3p)	Diagnostic biomarker for postmenopausal osteoporotic fragility fracture vs non-fracture control	Human	Serum-exosomes
	Shuai et al. (93)	5 miRNA panel: miR-30c-2-3p, miR-199a-5p, miR-424-5p, miR-497-5p, miR-877-3p 4 miRNA panel: miR30c-2-3p, miR-877-3p, miR-199a-5p, miR-424-5p	HIF1a pathway (miR-199a-5p) *RUNX2* (miR-30c) BMP signaling pathway (miR-497) Smad7 signaling (miR-877-3p)	Diagnostic biomarker for osteoporosis vs osteopenia and control	Human	Serum
	Weigl et al. (107)	miR-34a-5p, miR-31-5p, miR-30d-3p, miR-378a-5p (teriparatide and zolendronate) miR-375-3p, miR-183-5p, miR-203a-3p, miR-203b-3p (teriparatide)	*AGO3, MYC, SPRED1, MYCN, SON, NUFIP2, MDM4* Wnt signaling pathway Notch signaling pathway	Monitoring biomarkers for teriparatide and zoledronate treatment response in ovariectomized Sprague-Dawley rats vs control	Rat	Serum
	Xu et al. (108)	miR-491-5p miR-485-3p		Diagnostic biomarker for postmenopausal osteoporotic vertebral fracture vs postmenopausal controls without fracture	Human	Plasma
	Xu et al. (109)	miR-27a-3p	*SP7*	Diagnostic biomarker for osteoporosis vs controls	Human	Serum
	Yavropoulou et al. (110)	miR-124-3p miR-2861 miR-21-5p miR-23a-3p miR-29a-3p	*SPRY, PDCD4* (miR-21) *RUNX2* (miR-23-3p)	Diagnostic biomarker for postmenopausal osteopenic/osteoporotic vertebral fractures vs postmenopausal controls without fracture	Human	Serum
	Zarecki et al. (111)	miR-375 miR-532-3p miR-19b-3p miR-152-3p miR-23a-3p miR-335-5p miR-21-5p	*ESR1, ADCY1, ATF2, CALM1, PIK3R3, GNAQ, PIK3CA* (miR-19b-3p) *ITGA9, ITGA5, ITGA11, COL2A1, COL4A1, JARID2, INHBB, APC, WNT10B, IGF1, KLF4, MEIS1* (miR-152-3p) *YAP1, SMAD7, LATS1, BMPR2* (miR-21-5p)	Diagnostic biomarker for postmenopausal osteoporotic vertebral fracture vs postmenopausal controls without fracture	Human	Serum
	Zhao et al. (84)	miR-144-5p miR-506-3p miR-8068 miR-6841-3p	*YY1, VIM, YWHAE*	Diagnostic biomarker for PMO vs control	Human	Serum

OsteomiR™ panel: let-7b-5p, miR-127-3p, miR-133b, miR-141-3p, miR-143-3p, miR-144-5p, miR-152-3p, miR-17-5p, miR-188-5p, miR-19b-3p, miR-203a, miR-214-3p, miR-29b-3p, miR-31-5p, miR-320a, miR-335-5p, miR-375, miR-550a-3p, miR-582-5p; PMO: postmenopausal osteoporosis

Other authors sought to find biomarkers enabling diagnosis of osteoporotic fragility fractures. Previous studies aimed at identifying miRNA-biomarkers differentiating between patients with osteoporotic fragility fractures and patients without fractures (79, 85, 100, 106, 110, 111), patients with osteoporotic fractures versus (vs) patients with non-osteoporotic fractures (99, 101) and patients with osteoporotic fractures vs osteoarthritic controls without fractures (105) (**Table 1**).

Few authors studied a population over time in a prospective study to investigate potential predictive biomarkers for osteoporotic fragility fracture. Heilmeier et al. (97) assessed 168 postmenopausal diabetic women over a mean follow-up of 5.8 ± 2.7 years. They demonstrated increased fracture risk in women with upregulated miR-203a and miR-31-5p serum levels, whereas low expression of miR-19b-1-5p was associated with lower fracture risk. The predictive potential of this 3 miRNA-signature was increased when combined with BMD measurements (97). Ladang et al. (102) evaluated the predictive potential of a signature set of 19 miRNAs in a longitudinal study with an osteopenic/osteoporotic patient cohort. They successfully demonstrated a positive predictive value of 68% and a sensitivity of 76% in predicting fragility fracture within three years before the event occurred. They concluded that the miRNAs panel might be more valuable than the ordinarily used FRAX (Fracture Risk Assessment Tool) in predicting fragility fractures (102) (**Table 1**).

In a clinical setting, a monitoring biomarker may enable us to evaluate the status of treatment response. In 2020, Kocijan et al. (80) demonstrated upregulation of miR-203a in femoral head tissues and peripheral blood of ovariectomized Sprague-Dawley rats, which was reverted after 12 weeks of either resorptive treatment with zoledronate or osteo-anabolic treatment with teriparatide. Thus, besides being a diagnostic biomarker differentiating between ovariectomized rats and controls, miR-203a may also be a potential minimally invasive monitoring biomarker for treatment response (80). In 2021, the same group investigated the time dependent changes of miRNA serum expression levels during treatment in the same animal model (107). They presented two panels of miRNA biomarkers, miR-34a-5p, miR-31-5p, miR-30d-3p, miR-378a-5p and miR-375-3p, miR-183-5p, miR-203a-3p, miR-203b-3p, with upregulated serum expression levels over time, which was prevented by treatment with teriparatide or zoledronate and zoledronate only, respectively. In contrast to their previous study, the authors did not include miR-203a-3p in the zoledronate treatment response panel after demonstrating that the miR-203a-3p expression initially decreased during treatment, however, it increased again starting at 16 weeks of treatment (107). Ma et al. (82) investigated the biomarker potential of circulating miR-181c-5p and miR-497-5p, which were upregulated in the osteoporotic/osteopenic patient cohort vs healthy controls but decreased after anti-osteoporotic treatment with bisphosphonate and calcitriol, thereby representing a potential monitoring biomarker for treatment response. To assess denosumab treatment response in postmenopausal women, Messner et al. (104) demonstrated upregulation of a panel of 7 miRNAs, measured repeatedly over a period of 2 years, which was associated to increasing BMD and thereby representing a valuable monitoring biomarker set (**Table 1**).

In most studies, miRNAs are evaluated after onset of a disease. Interestingly, Quillen et al. (98) investigated the expression levels of 5 miRNAs in a cohort of 147 healthy adult baboons. All miRNAs were negatively correlated with BMD, thus increased miRNA levels correlated with decreased BMD. Target prediction highlighted their role in extracellular matrix regulation, apoptosis and cell proliferation. The authors suggest the investigated miRNA panel might be indicative of a pre-metabolic shift in bone homeostasis, thus showing promise as a susceptibility biomarker for development of osteoporosis (98) (**Table 1**).

Using a panel of biomarker vs single biomarkers increases specificity and sensitivity and allows for some variation, thus current trends in biomarker potential of miRNA go towards identification of disease-signature sets. Seeliger et al. (92) were among the first to identify a signature panel of circulating miRNA able to differentiate between osteoporotic and non-osteoporotic patients. They first performed miRNA PCR arrays in serum samples from patients with hip fractures, which were further classified in osteoporotic and non-osteoporotic sample. They identified 83 miRNAs, which were validated in a separate analysis using serum samples from osteoporotic and non-osteoporotic patients. Amongst those, 9 miRNAs were significantly upregulated in osteoporotic conditions. The authors proceeded in further validating these candidates in osteoporotic bone tissue. They conclude their study presenting a panel of 5 miRNAs (miR-21, miR-23a, miR-24, miR-25, miR-100, miR-125b) accurately diagnosing osteoporosis (92). The company TAmiRNA developed an osteomiR™ kit, that allows measurements of serum/plasma expression levels of 19 miRNAs, that have been extensively tested and validated in bone pathophysiology and bone-related diseases (80, 89, 100, 105, 111, 112). Kerschan-Schindl et al. (79) identified 4 clusters of miRNAs within the osteomiR™ set. Combining individual miRNA from different sets was shown to be relevant to the classification of osteoporosis according to the WHO definition and fracture-based classification (Major osteoporotic Fractures MOFx), with miR-375 as a main contributor for the former and miR-203 for the latter, respectively (79). Kocijan et al. (101) isolated a set of 19 miRNAs in osteoporotic patients vs healthy controls. Within the set, a panel of 8 miRNAs (miR-152-3p, miR-30e-5p, miR-140-5p, miR-324-3p, miR19b-3p, miR-335-5p, miR-19a-3p, miR-550a-3p) represent potential diagnostic biomarkers discriminating between patients with osteoporotic fractures vs non-osteoporotic fracture controls, regardless of age or sex. 7 of the 19 tested miRNAs are included in the osteomiR™ signature (**Table 1**).

## 4 MiRNA biomarkers in osteosarcoma

Osteosarcoma is the most common primary bone tumor occurring predominantly in children and young adults. Primary sites include the metaphyses of long bone, *e.g.* distal femur, proximal tibia or proximal humerus. By the time of diagnosis, about 15-25% of patients present with distant metastasis mostly to the lung, but also in bone and rarely in lymph nodes. Symptoms include local pain, swelling and limitation of movement. Diagnostic methods rely on traditional x-ray imaging techniques, often accompanied by magnetic resonance imaging (MRI) to detect soft tissue invasion and skip lesions, followed by bone tissue biopsy (113–116). Treatment requires a multidisciplinary approach including combination chemotherapy and complete surgical resection. Osteosarcoma is an aggressive tumor with a 5-year survival rate of 60-70%, which is drastically reduced to 10-40% in patients with relapsing disease or metastatic status at time of diagnosis (114, 117). Due to the difficulty in early assessment of relapse or diagnosis of minimal residual disease by conventional imaging techniques, biomarkers for monitoring of tumor response and surveillance of recurrence are highly sought after (115).

Multiple authors identify singular miRNAs as diagnostic biomarkers differentiating between osteosarcoma patients and a control group (118–141). Selection of miRNAs is based on previously established associations to different cancer types (118, 120–128, 131, 133, 135, 136, 139), which is an approach that drastically limits the potential candidate miRNAs. Other authors have focused on more comprehensive screening methods for potential biomarker selection, followed by validation by quantitative RT-PCR (138, 142, 143). Using this approach, Fujiwara et al. (142) demonstrated superiority of miR-25-3p as a diagnostic biomarker compared to the conventional biomarker ALP. Other authors created miRNA-mRNA regulatory networks using pre-screened miRNAs and their predicted targets. Candidate miRNAs were selected based on targeted hub genes [miR-199a-5p (137)] (**Table 2**).

**Table 2:** MiRNA biomarkers in osteosarcoma.

Bone-related disease	Author	MiRNA	Target genes of interest	Type of biomarker	Species	MiRNA source
**Osteosarcoma**	Allen-Rhoades et al. (115)	Panel: miR-205-5p, miR-335-5p, miR-574-3p, miR-214	*LZTS1* (miR-214) Wnt-signaling pathway, *SOX9* (miR-335-5p, miR-574-3p)	Diagnostic biomarker (all) Prognostic biomarker for overall survival (miR-214) Monitoring biomarker for disease development (all)	Mouse and human	Plasma
	Asano et al. (1)	Panel: Index VI (miR-4736, miR-6836-3p, miR-4281, miR-762, miR-658, miR-4649-5p, miR-4665-3p)		Diagnostic biomarker for sarcomas (including bone sarcomas)	Human	Serum
	Cao et al. (118)	miR-326	*BCL2*	Diagnostic biomarker Prognostic biomarker for distant metastasis	Human	Serum
	Cong et al. (119)	miR-124		Diagnostic biomarker Prognostic biomarker for overall survival	Human	Serum
	Cuscino et al. (116)	8 novel candidate miRNA		Diagnostic biomarker	Human	Osteosarcoma cell line-derived exosomes
	Dailey et al. (144)	Panel: miR-23a-3p, miR-30c-5p		Prognostic biomarker for disease-free interval	Canine	Serum
	Diao et al. (120)	miR-22		Diagnostic biomarker Prognostic biomarker for outcome (large tumor size, advanced clinical stages, distant metastasis) Predictive biomarker for chemosensitivity	Human	Plasma
	Dong et al. (121)	miR-223		Diagnostic biomarker Prognostic biomarker for distant metastasis, advanced clinical stage and overall survival	Human	Serum
	Fujiwara et al. (142)	miR-25-3p		Diagnostic biomarker Prognostic biomarker for distant metastasis	Human Mouse	Serum
	Gong et al. (145)	miR-675	*CALN1*	Prognostic biomarker for distant metastasis	Human	Plasma
	Heishima et al. (146)	Panel: miR-214, miR-126 and ALP		Prognostic biomarker for metastasis, disease free survival and overall survival	Canine	Plasma
	Hong et al. (147)	miR-29a miR-29b		Prognostic biomarker for disease free survival and overall survival	Human	Serum
	Hua et al. (122)	miR-Let7A		Diagnostic biomarker Prognostic biomarker for overall survival	Human	Blood
	Hua et al. (123)	miR-21	*PDCD4*	Diagnostic biomarker Predictive biomarker for chemosensitivity	Human	Serum
	Huang et al. (143)	miR-663		Diagnostic biomarker	Human	Plasma
	Huang et al. (148)	Panel: miR-487a, miR-493-5p, miR-501-3p, miR-502-5p)		Diagnostic biomarker	Human	Serum
	Jerez et al. (149)	miR-21-5p miR-143-3p miR-148a-3p miR-181-5p	*MAPK1, NRAS, FRS2, PRCKE, BCL2, QKI*	Prognostic biomarker for metastasis	Human	Osteosarcoma cell line-derived extracellular vesicles
	Kosela-Paterczyk et al. (150)	Panel: miR-133a, miR-223-3p, miR-450b-5p, miR-548q		Diagnostic biomarker	Human	Serum
	Li et al. (151)	miR-542-3p		Prognostic biomarker for tumor progression and overall survival	Human	Serum
	Lian et al. (152)	Panel: miR-195-5p, miR-199a-3p, miR-320a, miR-374-5p	*FASN* (miR-195-5p) p53 signaling pathway (miR-199-3p)	Diagnostic biomarker (4 miRNA set) Prognostic biomarker for metastasis (miR-195-5p, miR-199a-3p) Monitoring biomarker for tumor response after surgery	Human	Plasma
	Lian et al. (124)	miR-34a		Diagnostic biomarker Prognostic biomarker metastasis, recurrence and overall survival Predictive biomarker for chemosensitivity	Human	Serum
	Liu et al. (125)	miR-375		Diagnostic biomarker Prognostic biomarker for tumor stage and metastasis Predictive biomarker for chemosensitivity	Human	Serum
	Luo et al. (153)	miR-337-3p miR-484 miR-582 miR-3677		Diagnostic biomarker Prognostic biomarker for tumor stage, metastasis and overall survival Monitoring biomarker for surgical treatment response	Human	Serum
	Ma et al. (126)	miR-148a		Diagnostic biomarker Prognostic biomarker for tumor size, metastasis, overall survival and disease-specific survival	Human	Plasma
	Monterde-Cruz et al. (140)	miR-215-5p miR-642-5p	*RAB2A, RB1, BLCAP, CCNT2* (miR-215-5p) MAPK-signaling pathway, TGFß-signaling pathways (miR-642a-5p)	Diagnostic biomarker	Human	Serum
	Nakka et al. (141)	miR-21 miR-221 miR-106a	*RECK, PTEN* (miR-21) *HDAC6, DNMT3b, NOSTRIN, E-cadherin, uPAR7b, PTEN, KIP1, CDKN1B* (miR-221)	Diagnostic biomarker	Human	Plasma
	Niu et al. (127)	miR-95-3p		Diagnostic biomarker Prognostic biomarker for overall survival	Human	Serum
	Ouyang et al. (154)	Panel: miR-21, miR-199a-3p, miR-143		Diagnostic biomarker	Human	Serum
	Pang et al. (128)	miR-497		Diagnostic biomarker Prognostic biomarker for advanced stage, distant metastasis Predictive biomarker for chemosensitivity	Human	Serum
	Shi et al. (129)	miR-194	Multiple oncogenic target genes, *e.g. HIF-1a, YAP1, AKT2*	Diagnostic biomarker Prognostic biomarker for advanced clinical stage, metastasis and overall survival Monitoring biomarker for treatment response (surgery)	Human	Serum
	Tian et al. (130)	miR-34b		Diagnostic biomarker Prognostic biomarker for metastasis	Human	Plasma
	Wang et al. (131)	miR-491	*CRYAB*	Diagnostic biomarker Prognostic biomarker for lung metastasis and overall survival Predictive biomarker for chemosensitivity	Human	Serum
	Wang et al. (132)	miR-191		Diagnostic biomarker Prognostic biomarker for advanced clinical stage, distant metastasis, disease-free survival and overall survival	Human	Serum
	Xie et al. (133)	miR-26a-5p	*ITGB8, HOXA5*	Diagnostic biomarker Prognostic biomarker for advanced stage, metastasis and overall survival	Human	Serum
	Yang et al. (155)	miR-429 miR-143-3p		Diagnostic biomarker Prognostic biomarker for advanced stage, distant metastasis and overall survival	Human	Serum
	Yang et al. (134)	miR-221	*RECK, ARHI, DVL2*	Diagnostic biomarker Prognostic biomarker for distant metastasis, advanced clinical stage, disease-free survival and overall survival	Human	Serum
	Yao et al. (135)	miR-101	*RAC1*, *USP22, VEGF-C*, Girdin, *COX-2, EZH2, SOCS-2, ZEB1, ZEB2*	Diagnostic biomarker Prognostic biomarker for advanced clinical stage, distant metastasis, disease-free survival and overall survival Monitoring biomarker for treatment response in non-metastatic patients	Human	Serum
	Yuan et al. (136)	miR-21		Prognostic biomarker for advanced stage and overall survival Predictive biomarker for chemosensitivity	Human	Serum
	Zhang et al. (156)	miR-133b miR-206	*EGFR, MCL1, FSCN1*, c-Met*, BCL2L2* (miR-133b)	Diagnostic biomarker Prognostic biomarker for high tumor grade, metastasis, recurrence, disease-free survival and overall survival	Human	Serum
	Zhang et al. (137)	miR-199a-5p	*VEGFA*	Diagnostic biomarker	Human	Osteosarcoma cell line-derived exosomes Plasma
	Zhou et al. (138)	miR-199a-5p		Diagnostic biomarker Monitoring biomarker for surgical treatment response and tumor status	Human	Serum
	Zhou et al. (139)	miR-421		Diagnostic biomarker Prognostic biomarker for overall survival	Human	Serum

High biovariability and small sample sizes due to the rarity of the disease make biomarker identification in osteosarcoma difficult. Multiple authors have focused on multi-panel miRNA signatures to increase biomarker accuracy and allow for individual variations (1, 115, 116, 148, 150, 152–154). Asano et al. (1) screened serum miRNAs in over a 1000 sarcoma samples, including osteosarcoma. They identified a classifier Index VI differentiating between sarcomas and benign tumors and healthy controls. The group validated their findings in a smaller subset of patients and also comparing to patients with other malignancies, *e.g.* lung cancer. Although the discriminatory ability of Index VI was high in bone sarcomas, especially in the early stages, the authors point out that the Index does not discriminate between histological subtypes and that without confirmatory studies, it should only be used for diagnosis of malignant vs benign tumors (1). In 2015, Allen-Rhoades et al. (115) developed a mouse model for osteosarcoma and chose a signature set of four candidate miRNAs differentiating between affected mice and their healthy controls. The authors validated their findings in human plasma samples and thereby demonstrated cross-species application of their model (115) (**Table 2**).

Additionally, multiple miRNAs have been suggested as prognostic biomarkers in osteosarcoma. High/low expression levels of specific miRNAs have been associated with advanced tumor stage, positive metastasis, higher recurrence and/or shorter overall survival (115, 124–136, 139, 141, 142, 144, 145, 147, 151, 152, 154–156). Similarly, multiple authors demonstrated superiority of using a miRNA panel with or without combination with clinical markers for prognostic accuracy compared to individual miRNAs (146, 149, 153) (**Table 2**).

Monitoring biomarkers in osteosarcoma are useful to assess adequate response to surgical resection or chemotherapy and to screen for recurrence. Some authors identified upregulated miRNA expression levels preoperatively, that decreased upon surgical tumor resection (138, 142, 152) and vice versa, respectively (129, 135, 153). Considering that these miRNAs react to changes in tumor status, they represent valuable candidates for monitoring surgical treatment response and detecting residue and recurrence. In addition to surgery, combination chemotherapy is required for a curative approach in treating osteosarcoma (114). Fujiwara et al. (142) evaluated the potential of miR-25-3p to monitor tumor response in a case series and were able to show decreasing expression levels after surgical tumor resection and during/after neoadjuvant combination chemotherapy, unlike ALP levels, which plateaued during treatment. In a mouse model of osteosarcoma, Allen-Rhoades et al. (115) demonstrated detectable changes in expression levels of a previously identified 4-miRNA signature set coinciding with tumor formation 14 weeks after transplantation of osteosarcoma cells. Additionally, they measured expression level of a 4-miRNA set in placebo treated and Doxorubicin-treated mice with osteosarcoma and were able to demonstrate reduced magnitude of alteration in miRNA plasma expression levels in the treated mice, suggesting that these miRNAs may be a valuable monitoring marker for tumor development and chemo-response (**Table 2**).

Predictive biomarkers may guide treatment decisions in osteosarcoma as certain preconditions, *e.g.* specific miRNA up- or downregulation, may indicate the likelihood of a patient to respond well or poorly to a chemotherapy. For instance, patients with lower levels of certain miRNAs before chemotherapy, such as miR-34 (124) demonstrated good treatment response compared to patients with initially high miRNA expression levels. In contrast, patients with low levels of miR-375 (125), miR-497 (128), miR-491 (131) or miR-21 (136) before neoadjuvant chemotherapy showed a poor treatment response (**Table 2**).

## 5 MiRNA biomarkers in other bone-related diseases

Besides osteosarcoma, Ewing Sarcoma is the second most common bone and soft tissue tumor in children and adolescents. The Ewing’s sarcoma family of tumors (ESFT) includes Ewing’s sarcoma, Askin tumor and peripheral primitive neuroectodermal tumor. At the time of diagnosis, about 25% of patients have detectable metastasis, primarily in the lung, bone and bone marrow. Treatment includes combination chemotherapy and surgical resection (157). Using miRNA sequencing, Crow et al. (158) identified a disease-specific signature set of 62 exosomal miRNAs able to differentiate between ESFT and non-ESFT samples. Similarly, Kosela-Patercyzk et al. (150) and Nie et al. (159) identified a 4 miRNA panel and a singular miRNA marker, respectively, as diagnostic biomarkers for ESFT. In chondrosarcoma, a type of bone sarcoma, miR-145 (160) was identified as a diagnostic biomarker for early detection, which is important due to limited treatment options. However, studies investigating miRNA biomarkers for these types of bone sarcomas are rare (**Table 3**).

**Table 3:** MiRNA biomarkers in other bone-related diseases.

Bone-related disease	Author	MiRNA	Target genes of interest	Type of biomarker	Species	MiRNA source
**Osteoarthritis**	Ali et al. (161)	miR-320b miR-320c miR-320d miR-320e	14-3-3 gene family	Diagnostic biomarker for fast-progressing OA	Human	Plasma
	Pertusa et al. (162)	miR-497	*SMURF2*	Diagnostic biomarker	Human	Serum
	Wan et al. (163)	miR-136	*IL17*	Diagnostic biomarker	Human	Plasma
	Xia et al. (164)	miR-181-5p	*TNFA*	Diagnostic biomarker	Human	Peripheral blood
**Ewing Sarcoma Family of Tumors (ESFT)**	Crow et al. (158)	Panel of 62 miRNAs (see full list in original paper)		Diagnostic biomarker	Human	ESFT cell line-derived extracellular vesicles Serum
	Kosela-Paterczyk et al. (150)	Panel: miR-424-5p, miR-3173-3p, miR-142-3p, miR-4746-5p		Diagnostic biomarker	Human	Serum
	Nie et al. (159)	miR-125b		Diagnostic biomarker	Human	Serum
**Chrondrosarcoma**	Urdinez et al. (160)	miR-145	*FSCN1*	Diagnostic biomarker	Human	Plasma
**Multiple Myeloma Bone Disease**	Papanota et al. (165)	Panel: let-7b-5p, miR-143-3p, miR-17-5p miR-214-3p, miR-335-5p	*ATF4, SP7, FGFR1, PTEN* (miR-214-3p) *SP7* (miR-143-3p) *TGFBR1, IGF1R, MYC* (let-7a-5p) *DKK1, IGF1R* (miR-335-5p) *SMAD5, BMP2* (miR-17-5p)	Diagnostic biomarker Prognostic biomarker for progression-free survival (let-7b-5p, miR-335-5p)	Human	Plasma
	Moura et al. (166)	miR-29c-3p		Diagnostic biomarker	Human	Plasma
	Hao et al. (167)	miR-214 miR-135b	*PTEN* (miR-214)	Prognostic biomarker for bone disease in Multiple Myeloma patients Predictive biomarker for bisphosphonate treatment response (miR-214)	Human	Serum
**Bone metastasis**	Fang et al. (168)	miR-214	*PTEN*	Prognostic biomarker for bone metastasis in PCa	Human	Serum
	Guo et al. (169)	miR-205		Prognostic biomarker for bone metastasis in PCa	Human	Serum
	Lu et al. (170)	miR-125a-3p miR-330-3p miR-339-5p miR-613	*KRAS, RHOA, BDNF, HNRNPA1, BRCA1, FYN, BCL2L11, NRG1, SETX, FXR1, BACE1, CDC37, TIMP3, PDCD4, SGK1*	Prognostic biomarker for bone metastasis in PCa	Human	Plasma-derived exosomes
	Peng et al. (171)	miR-218-5p	*TRAF1, TRAF2, TRAF5*	Prognostic biomarker for bone metastasis in PCa	Human	Serum
	Rode et al. (172)	miR-425-5p	*HSPB8*	Prognostic biomarker for bone metastasis in PCa	Human	Prostate cancer cell-line derived exosomes
	Wa et al. (173)	miR-204-5p	*TRAF1, TAB3, MAP3K3*	Prognostic biomarker for bone metastasis in PCa	Human	Serum
	Wang et al. (174)	miR-181-5p		Prognostic biomarker for bone metastasis in PCa	Human	Serum-exosomes
	Zhang et al. (175)	miR-141		Prognostic biomarker for bone metastasis in PCa	Human	Serum
	Wu et al. (176)	miR-19a	*PTEN*	Prognostic biomarker for bone metastasis in breast cancer	Human	Bone-metastatic estrogen-receptor positive breast cancer cell-derived exosomes
	Zhao et al. (177)	miR-10b		Prognostic biomarker for bone metastasis in breast cancer	Human	Serum
	Xiang et al. (178)	miR-34a	*TGIF2*	Prognostic biomarker for bone metastasis in hepatocellular carcinoma	Human	Serum
	Valencia et al. (179)	miR-326		Monitoring biomarker for metastatic progression in bone metastasis in lung cancer	Mouse	Serum
	Xu et al. (180)	miR-139-5p	*NOTCH1*	Prognostic biomarker for bone metastasis in lung cancer	Human	Serum
	Yang et al. (181)	Panel of 144 miRNA (Cluster B, see (181)), *e.g*. miR-574-5p, miR-328-3p, miR-423-3p	Wnt/ß-catenin signaling pathway	Prognostic biomarker for bone metastasis in lung cancer	Human	Plasma-derived exosomes
	Zeng et al. (182)	miR-31-3p	*FOXO1*	Prognostic biomarker for bone metastasis in lung cancer	Human	Serum
**Fracture healing**	Bourgery et al. (183)	miR-451 miR-328-3p miR-133a-3p miR-375-3p miR-423-5p miR-150-5p	*P38 MAPK* (miR-451) *Axin1, PTEN* (miR-328-3p) *Egfr, Fgfr1, Igfr1, Met* (miR-133a-3p) *Lrp5, Ctnnp, Brd4* (miR-375-3p) *Tnip2, Cdkn1a, Igf2bp1* (miR-423-5p) *Vegf, Socs1, Rab9, Mmp14, Slc2a1, Elk1* (MiR-150-5p)	Monitoring biomarker for adequate fracture healing	Mouse	Serum
	Dietz et al. (184)	miR-223		Prognostic biomarker for development of CRPS after fracture	Human	Serum-exosomes
	Xiong et al. (185)	miR-193a-3p	*MAPK10*	Prognostic biomarker for fracture non-union	Human	Serum
**Adolescent idiopathic scoliosis**	García-Giménez et al. (186)	Panel: miR-122-5p, miR-27a-5p, miR-223-5p, miR-1306-3p	Wnt/ß-catenin pathway, *BMP4* (miR-122-5p) *APC, PPARG, CEBPA* (miR-27a) *FGF2, PCGF3, BMI1, PU.1, RANKL, NFATc1, TRAP*, c-Jun, cathepsin K, *NFIA* (miR-223-5p)	Diagnostic biomarker	Human	Plasma
	Wang et al. (187)	miR-151a-3p	*GREM1*	Diagnostic biomarker	Human	Plasma
**Osteonecrosis of the femoral head (ONFH)**	Hong et al. (188)	miR-127-3p miR-628-3p miR-1 miR-885-5p miR-483-3p miR-483-5p	*IGF2, PDGFA, RUNX2, PTEN, VEGF*	Diagnostic biomarker for alcohol-induced ONFH	Human	Serum
	Kao et al. (189)	miR-18a miR-19a miR-138-1 miR-1290 miR-3609	*TP53* *SERBP1*	Diagnostic biomarker	Human	Blood
	Liu et al. (190)	miR-93-5p miR-320a		Diagnostic marker	Human	Serum
**Fibrous dysplasia of the bone**	Legrand et al. (191)	miR-25-3p miR-93-5p miR-182-5p miR-324-5p miR-363-3p miR-451a	*IL6ST* (miR-25-3p, miR-363-3p) *FOXO1* (miR-324-5p) *IL6R* (miR-451a) *GNAS, FOXO3, PDGFB, ESR2, BMP2* (miR-93-5p)	Diagnostic biomarker	Human	Serum
**Developmental dysplasia of the hip**	Luo et al. (192)	miR-140 and 25-hydroxyvitamin D status		Diagnostic biomarker	Human	Serum
**Renal osteodystrophy**	Nickolas et al. (193)	Panel: miR-30b, miR-30c, miR-125b, miR-155		Diagnostic biomarker low vs non-low bone turnover	Human	Serum

Multiple Myeloma is a hematological malignancy of the bone marrow. One of the more severe complications of Multiple Myeloma is bone disease, which is defined by osteolytic lesions or osteoporotic fractures due to clonal plasma cell disorder. Even though imaging techniques are improving quickly, early diagnosis of bone disease using non-invasive techniques would be very beneficial to guide treatment decisions (194). To our knowledge, three studies have investigated circulating miRNA as biomarkers for multiple myeloma bone disease, using a 5 miRNA-panel (165), miR-29c-3p (166), and miR-214 and miR-135b (167) as individual biomarkers, respectively. Out of the 5 miRNA-panel, two miRNAs (let-7b-5p and miR-335-5p) were also identified as a prognostic biomarker for progression-free survival (165). Hao et al. (167) identified miR-214 as a predictive biomarker for good response to bisphosphonate treatment (**Table 3**).

Bone homeostasis is maintained by a constant remodeling process and relies on a balance of osteoblast and osteoclast activity. Solid tumors often metastasize to bone and over 70% of metastatic prostate and breast cancer patients experience bone metastasis. Tumor cells mainly infiltrate the endosteal and perivascular niches and are thought to remain in a state of dormancy often up to many years. Upon activation of tumor cells, cross-talk between the bone modulating cells leads to a release of growth factors and tumor cell proliferation is promoted by a feed-forward loop. Symptoms of bone metastasis include pain and increased fracture risk. Tumors are often considered incurable once bone metastasis has occurred and current diagnostic methods expose patients to radiation, thus biomarkers for early detection and prediction of patients at risk are highly sought after (176, 195). Multiple miRNA biomarkers have been identified for detection of bone metastasis in prostate cancer (168–175), breast cancer (176, 177), hepatocellular carcinoma (178) and lung cancer (180–182). Additionally, in lung cancer bone metastasis, Valencia et al. (179) identified miR-326 as a valuable biomarker to monitor metastatic progression and tumor burden. It correlated with the conventional bone turnover marker PINP (procollagen I amino-terminal pro-peptide (179) (**Table 3**).

Osteoarthritis is one of the leading causes of disability in adults worldwide and is a socioeconomic and financial burden. It is a metabolic inflammatory disease that leads to progressive cartilage degeneration. Major symptoms include chronic pain, stiffness and loss of mobility (196). MiRNAs have been investigated as diagnostic biomarkers in osteoarthritis (162–164). Additionally, Ali et al. (161) has identified the miR-320 family as potential diagnostic biomarker for fast-progressing osteoarthritis.

Long-bone fracture healing occurs in stage starting with hematoma formation, inflammation, migration and differentiation of mesenchymal stromal cells (MSCs), bone formation and angiogenesis and bone remodeling. During endochondral ossification, hypertrophic chondrocytes turn into osteoblasts. This process is tightly controlled by miRNAs (183). Any disruption of this complex process can lead to fracture non-union, arthrosis and chronic pain syndromes. Bourgery et al. (183) analyzed the serum expression levels of circulating miRNAs during fracture healing in a mouse model. They identified differentially expressed miRNAs over a follow-up period of 14 days compared to the baseline and were able to establish a potential miRNA signature for monitoring adequate fracture healing (183). Similarly, Xiong et al. (185) demonstrated miR-193a-3p as a potential prognostic biomarker indication fracture non-union. Development of chronic regional pain syndrome (CRPS) after fracture is often difficult to diagnose with potentially devastating consequences for the patient. Dietz et al. (184) identified miR-223-5p as a potential prognostic biomarker for development of CRPS after bone fracture (**Table 3**).

Other bone-related diseases that have been studied in the context of circulating miRNA expression levels include adolescent idiopathic scoliosis (186, 187), osteonecrosis of the femoral head (188–190), fibrous dysplasia of the bone (191), developmental dysplasia of the hip (192) and renal osteodystrophy (193) (**Table 3**).

## 6 Discussion

This review discusses the potential use of circulating and EV-derived miRNA as biomarkers of bone-related disease. Although this is a promising approach for diagnostic, monitoring, prognostic and predictive purposes, this area of research is still in early stages and has many limitations.

One of the main criticisms of circulating miRNAs as biomarker of disease is its lack of reproducibility, low specificity and high intra- and inter-assay variability. In the studies discussed in this review, there is only minimal overlap of biomarker candidates in osteoporosis, even less so in osteosarcoma studies. Moreover, some studies are even directly contradictory, *e.g.* Yang et al. (134) demonstrated high levels of miR-221 associated with poor prognosis and Nakka et al. (141) reported the reversed situation. These inconsistencies are due to lack of standardization of pre-analytical and analytical conditions and unaccounted differences in patient population, *e.g.* sex, age and existing preconditions (53, 197). For instance, centrifugation conditions can impact measurements of miRNA expression in plasma or serum samples and differences in miRNA purification methods accounts for 77-92% of intra-assay variation in miRNA quantification (197). Additionally, detection methods for circulating miRNA include Next-Generation Sequencing (NGS), real-time PCR, miRNA microarray, and less frequently, Northern blot analysis and *in situ* hybridization (198). To assure consistency between studies, miRNA detection should be accompanied by an adequate normalization strategy. Usually, this can be done by using a reference marker, most commonly spike-in cel-miR-39 or endogenous miR-16 during RNA extraction. A combination of spike-in and endogenous reference markers is preferred (197–199). The osteomiR™ panel contains three spike-in controls as well as two endogenous reference markers for hemolysis (79). In osteosarcoma, Allen-Rhoades et al. (115) investigated cross-species application of miRNA biomarkers and identified disease-specific endogenous reference plasma miRNAs in mouse and human.

Moreover, selection of biomarker candidates is inconsistent and unreliable, leading to low specificity. Many authors do not perform comprehensive screening methods to select potential miRNA biomarker candidates, but rather rely on previously published literature selecting miRNAs that have been shown to play a role in related diseases. To identify novel candidates that are specific to the investigated disease, comprehensive screening methods need to be performed and potential biomarkers should be selected based on differential expression levels at multiple different time points and their known or potential biological and molecular function. For instance, miR-21 is a widely known oncogenic miRNA implicated in tumorigenesis of multiple entities. Overexpression of miR-21 has been demonstrated in multiple tumors, including glioblastoma, ovarian cancer, B-cell lymphoma, hepatocellular carcinoma, lung cancer and breast cancer and is associated with increased proliferation and invasion and decreased apoptosis (200, 201).Target genes include tumor suppressor genes, *e.g. PTEN*, programmed cell death 4 (*PDCD4*), *SMAD7* and tropomyosin (*TPM1*). Thus, miR-21 can be valuable as a monitoring biomarker for tumor progression or treatment response or as part of a diagnostic panel of miRNA, as suggested by many authors (136, 141, 149, 154), but its value as an individual diagnostic biomarker is limited (53).The Receiver Operating Characteristic (ROC) approach allows calculation of sensitivity and specificity and generation of an area under the curve (AUC) which is an indicator for how well the biomarker differentiates between a disease and its control (202). Many, but not all studies, discussed in this review specify AUC values and accepted cut-off values are not standardized and/or inconsistent, leading to low specificity of biomarker candidates (115). Additionally, according to the BEST Glossary by the FDA-NIH Biomarker Working Group, the test conditions play an important role in biomarker validation. For instance, a single measurement of blood measure is not sufficient for diagnosis of hypertension. The studies discussed in this review often measure expression levels of the candidate miRNA at only one defined time point. However, many miRNA expressions change with disease progression, which might explain some contradictions. Generally, given the heterogeneity of disease, using a panel compared to individual biomarkers is advisable (198). Further, positive and negative predictive values are dependent on the prevalence of disease in the population and these values were only rarely discussed in the presented studies (102, 151).

The majority of circulating biomarkers in blood is released by blood or endothelial cell and is thus not directly derivative of a specific diseased tissue, *e.g.* a tumor, and does not have biomarker potential. Even though it is well known, that tumors secrete miRNA packaged in EVs into the extracellular fluid, the fraction of these tumor-derived miRNAs compared to the larger fraction of the physiologically present endothelial/blood cell derived miRNAs is small and depends on the size of the tissue, access to variation and a large enough magnitude of differential expression compared to healthy tissue. Circulating miRNA biomarkers are thus not directly tumor- or disease-derivative, but rather a physiological response to the presence of a neoplastic formation or other disease. Moreover, miRNA expression levels vary greatly depending on sex, ethnicity, lifestyle and sample cell type (199). In contrast, EV-miRNAs are expressed stably and protected from degradation. Additionally, they express surface markers, that are highly specific to their tissue of origin. New isolation and purification methods allow for harvesting of EVs based on expression of surface marker proteins (53, 197–199). Hereby, EVs and their cargo could be directly linked to the releasing tissue and allow for differentiation of small disease-dependent miRNA from the larger fraction of the physiological miRNA secretome (53). Thus, EV-derived miRNA content is more tissue-specific and selective analysis harbors potential to improve the specificity of circulating EV-miRNA biomarker candidates.

## 7 Conclusions and future perspectives

In the era of “personalized/precision medicine”, discovery of new biomarkers takes a growing place. An ideal biomarker needs to be easily accessible, highly specific, and preferably sensitive in early detection of disease and changes in disease status due to progression or treatment (198). Given that circulating miRNA are secreted in extracellular fluids and are thereby easily harvested by minimally invasive liquid biopsy, are disease- and tissue-specific and reflective of even small changes in disease status, they technically meet the criteria of an ideal biomarker. However, technical limitations like lack of standardization of screening methods, selection and analysis leads to low reproducibility and severely undermines the value of the currently existing data. However, considering that this area of research is still in its early stages, adequate standardization of techniques, analysis and interpretation of results and comprehensive description of applied methods can help support large-scale validation studies of the promising, but preliminary, data known today. Additionally, selective analysis of EV-miRNA based on surface marker proteins represents a promising approach to further optimize specificity of the miRNA biomarker candidates.

## Author contributions

JH was responsible for conception of the study, data collection and analysis and drafting the manuscript. ML was responsible for conception of the study and manuscript drafting and revision. NQ was responsible for conception of the study, drafting the article and final revision. All authors contributed to the article and approved the submitted version.
